# Editorial: Misuse and abuse of prescription drugs in custodial settings

**DOI:** 10.3389/fpsyt.2023.1339542

**Published:** 2023-11-30

**Authors:** Filippo Pirani, Stefania Chiappini, Fabrizio Schifano, Arianna Giorgetti

**Affiliations:** ^1^Unit of Legal Medicine, Department of Medical and Surgical Sciences, University of Bologna, Bologna, Italy; ^2^School of Medicine, UniCamillus International Medical School University, Rome, Italy; ^3^Psychopharmacology, Drug Misuse, and Novel Psychoactive Substances Research Unit, School of Life and Medical Sciences, University of Hertfordshire, Hatfield, United Kingdom

**Keywords:** substance use, custodial setting, problematic drug use, abuse monitoring model, prisons /prisoners, psychiatric hospitals

Custodial settings, such as prisons and psychiatric hospitals, are meant to be places of rehabilitation and security. Beside the abuse of illicit drugs, a growing concern about these institutions is the misuse and abuse of prescription drugs. Prescription drug abuse is the use of a prescription medication in a way not intended by the prescriber. The consequences of this serious public health problem range from an increase in treatment and emergency room admission, to raise in addiction and overdose deaths (1). The most abused drugs generally include opioid-based painkillers, anti-anxiety medications, sedatives and stimulants (2–4). The easier way to obtain them in comparison to illegal drugs and the possibility to avoid controls might explain the spreading of prescription drug misuse and abuse in particular settings. However, due to a number of ethical challenges, first of all the need for safeguarding the free participation of people, the research in the custodial setting is still limited. This Research Topic explores this issue examining four papers.

A study conducted in German prisons by Franchetti et al. sheds light on the prevalence of illicit use of opioid substitution drugs, specifically methadone and buprenorphine. Out of 675 participants, 10.4% tested positive for methadone, 10.4% for buprenorphine, and 0.6% for both drugs. Alarmingly, at least 14.8% of participants tested positive to drug analyses were not prescribed opioid substitution treatment (OST), indicating an illicit use of the substances. The article highlights the challenges associated with opioid misuse in the German prison settings, including the risk for overdose, transmission of diseases like HIV and HCV, and potential complications due to intravenous drug administration. This study also underscores the need for strategies to prevent the illicit drugs supply into prisons and for measures to address untreated opioid use disorders among inmates.

A particular concern in relation to substance abuse arises from incarcerated older adults (IOAs). IOAs, indeed, represent a growing global population within custodial settings, and their experiences with drug use reveal complex interactions between aging, incarceration, and substance abuse. Four distinctive themes emerged from their drug-related experiences, as reported by the study of Avieli, highlighting the importance of tailored interventions and support for IOAs with substance abuse issues. Understanding these narratives can aid professionals in developing effective interventions to address their drug abuse and related challenges.

Prescription Medication Abuse (PMA) is perceived as a problem also in China, where it has been recognized the need for a tailored monitoring model for psychiatric hospitals. A survey involving 36 monitoring institutions and 358 medical staff revealed strong support for PMA monitoring in psychiatric hospitals. The study by Du et al. details the establishment of an active real-time monitoring model, which involves identifying PMA cases during diagnosis and treatment, prescription medication concentration detection, and laboratory screening. The results showed a significant increase in reported PMA cases, primarily among adolescents who abuse class II psychotropic drugs in order to self-manage anxiety and depression. This monitoring model is a promising step toward improving the management of prescription medications in psychiatric hospitals and contributes to the development of national PMA monitoring policies.

A peculiar custodial setting is the population convicted for driving under the influence (DUI) of substances. A systematic review by Giorgetti et al. examines the impact of co-consumption of ethanol and synthetic cannabinoids (SCs) on psychomotor performance relevant to driving, by also considering people in the custodial setting for DUI-related crimes. The review identifies a wide range of prevalence rates for co-consumption, highlighting the challenges associated with detecting SCs in standard drug tests. The synergistic effects on the central nervous system increase the risk of impaired driving performance, emphasizing the need for further research and tailored interventions to address this growing concern. Further research on the impact of co-consumption of drugs and particularly of illicit and prescription drugs within the custodial setting is needed.

The misuse and abuse of prescription drugs within custodial settings is a global concern that requires urgent attention. The summarized articles shed light on the prevalence of illicit prescription and also non-prescription drug use, the need for tailored monitoring models, the unique challenges faced by incarcerated older adults, and the attention needed on people convicted in driving under the influence.

To address these issues effectively, custodial institutions, healthcare professionals, politics and policymakers must work together to implement preventative measures, provide appropriate treatment and support, and enhance monitoring and reporting systems. Failure to do so not only jeopardizes the health and wellbeing of those in custodial settings but also poses risks to society as a whole. It is imperative that we recognize the gravity of this issue and take concrete steps to address it comprehensively, starting from a methodological approach to develop the research in a setting of vulnerable people.

## Author contributions

FP: Writing—original draft. SC: Writing—original draft. FS: Writing—original draft. AG: Writing—original draft.

## References

[1] Tay E Makeham M Laba TL Baysari M. Prescription drug monitoring programs evaluation: a systematic review of reviews. Drug Alcohol Depend. (2023) 247:109887. 10.1016/j.drugalcdep.2023.109887 37126936

[2] Ates Bulut E Isik AT. Abuse/misuse of prescription medications in older adults. Clin Geriatr Med. (2022) 38:85–97. 10.1016/j.cger.2021.07.004 34794705

[3] Schifano F Chiappini S Corkery JM Guirguis A. Abuse of prescription drugs in the context of novel psychoactive substances (NPS): a systematic review. Brain Sci. (2018) 8:73. 10.3390/brainsci8040073 29690558 PMC5924409

[4] Schifano F Chiappini S Miuli A Mosca A Santovito MC Corkery JM. Focus on over-the-counter drugs' misuse: a systematic review on antihistamines, cough medicines, and decongestants. Front Psychiatry. (2021) 12:657397. 10.3389/fpsyt.2021.657397 34025478 PMC8138162

